# Supplementary material for: A licensing step links AID to transcription elongation for mutagenesis in B cells
Source: Nat Commun. 2018 Mar 28;9:1248. doi: 10.1038/s41467-018-03387-6 (PMC5871760; doi:10.1038/s41467-018-03387-6)
Supplement: Supplementary file 1 — Supplementary Information [file 41467_2018_3387_MOESM1_ESM.pdf]

A licensing step links AID to transcription elongation for mutagenesis in B cells

Methot et al.

Supplementary information

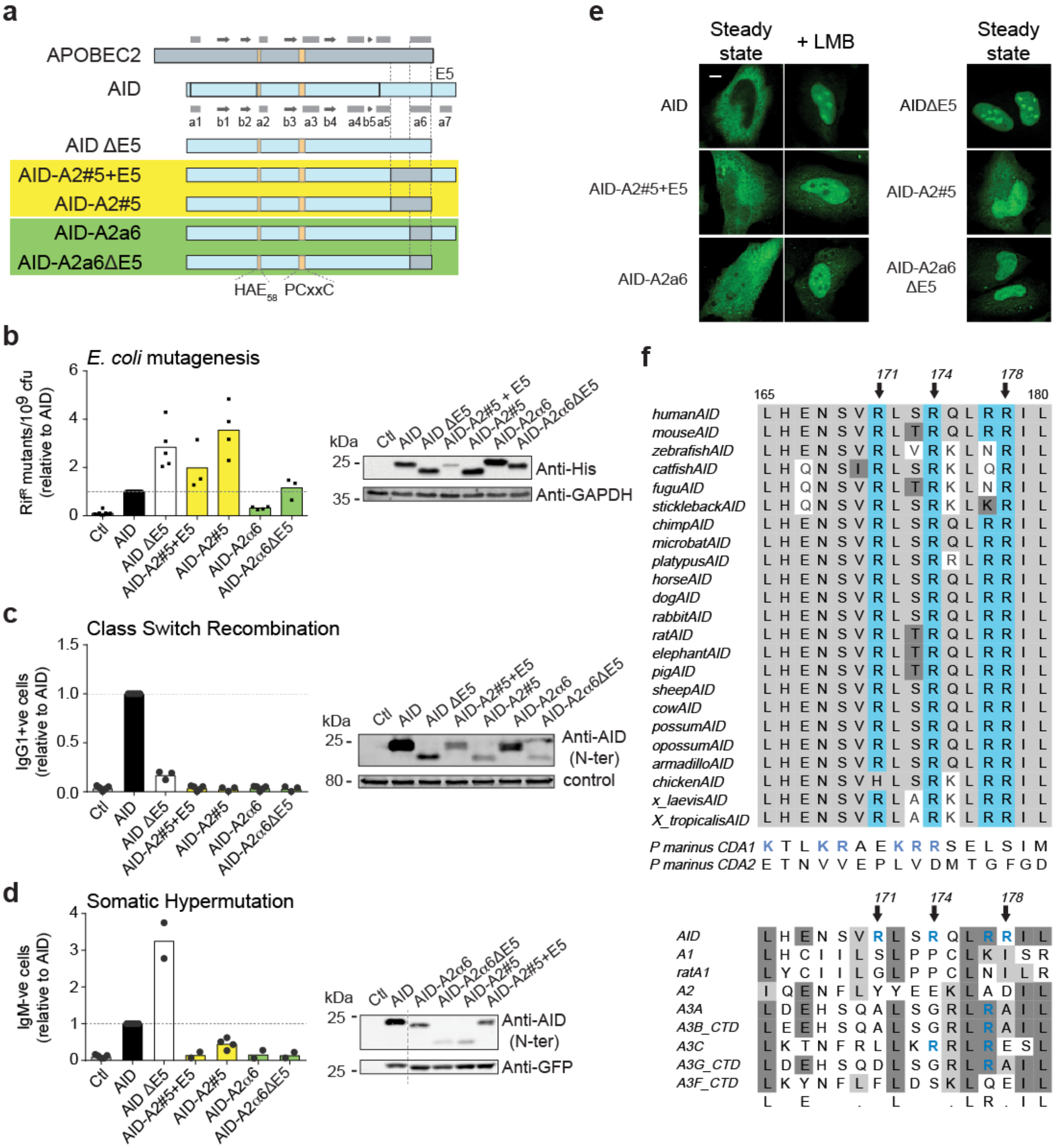

Supplementary Figure 1. The alpha helix 6 of AID is required for biological function.
(**a**) Schematics of APOBEC2 (A2), AID and AID-A2 chimeras. E5 denotes the region encoded by *AICDA* exon 5. Secondary structure elements, α-helices (light grey rectangles) and β-sheets (dark grey arrows) are identified. (**b**) Mutagenic activity in E. coli measured by the frequency of rifampicin resistant (Rif^R) colonies (caused by mutations in *RpoB)* arising from cultures expressing AID or the chimeras. Means (bars) of median values (dots) obtained from 3-5 independent experiments (5 cultures/experiment) are shown, normalized to AID. (**c**) Class switch recombination activity in $Aicda^{-/-}$ mouse primary B cells transduced with AID or the indicated chimeras -ires-GFP and stimulated with LPS and IL-4. Means (bars) proportion of IgG1+ cells in the GFP+ population 72 h after transduction 3-5 independent experiments are shown (dots indicate values of each individual mouse), normalized to AID. (**d**) Somatic hypermutation activity was assayed by the relative IgM-loss accumulation in cultures of DT40 $Aicda^{-/-}$ ΔΨVλ B cells complemented with AID or the indicated chimeras -ires-GFP. Means (bars) of the median values (dots) obtained from 2-4 independent experiments (≥12 cultures/experiment) were normalized to the mean value of AID. In (**b-d**) WB of cell extracts probed with anti-AID antibody and loading control are shown on the right. For gel source data see supplementary Fig. 7. (**e**) Representative confocal microscopy images of HeLa cells transiently expressing AID and chimeras fused to GFP under steady state or after nuclear export inhibition with LMB (50 ng/mL, 2h). Representative of 2 independent experiments. Magnification 400X. Scale bar, 10 µm. (**f**) Alignment of amino acid sequence of the region corresponding to the α6 helix of AID from multiple vertebrate species (top) or the α6 helix of AID and various APOBECS (bottom). Arg 171, 174 and 178 residues are indicated and basic residues at those positions are highlighted in blue.

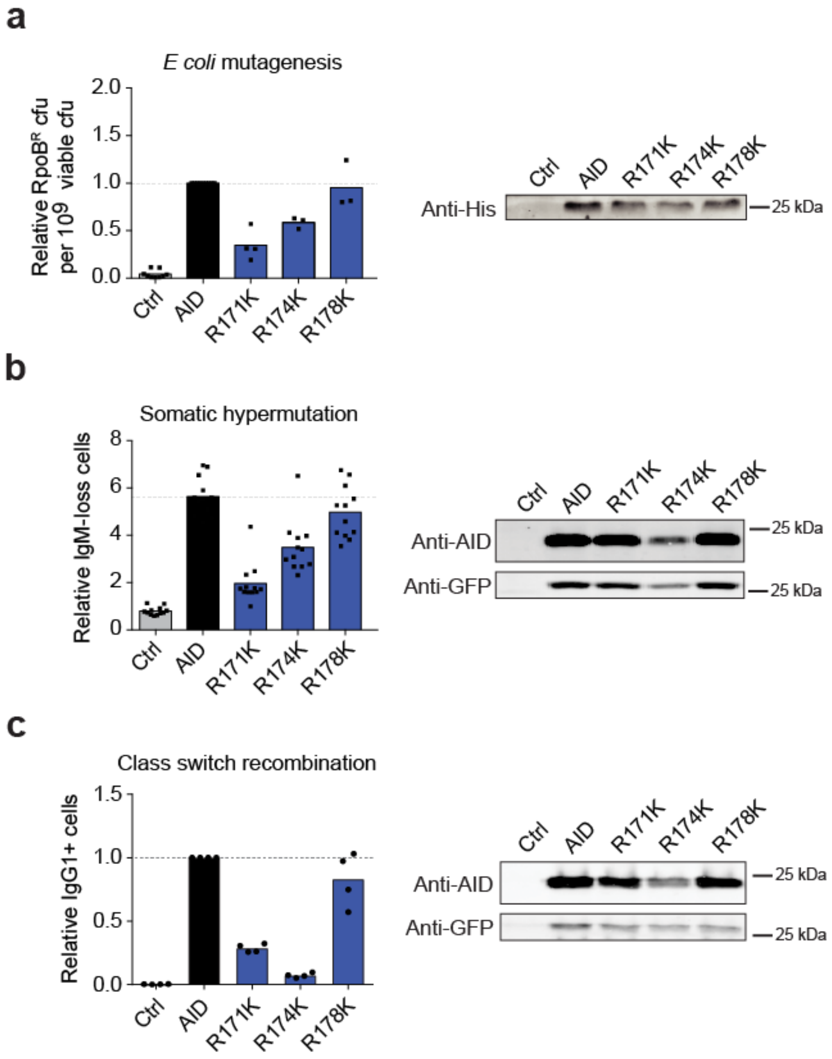

Supplementary Figure 2. Distinct contribution of Arg 171, 174 and 178 to AID function.
(**a**) Mutagenic activity in *E. coli* measured by the frequency of Rif resistant colonies arising from cultures expressing AID variants or empty vector (Ctrl). Means (bars) of median values (dots) obtained from 3-4 independent experiments (5 cultures/experiment) are shown, normalized to AID. (**b**) Somatic hypermutation activity was assayed by the relative IgM-loss accumulation in cultures of DT40 *Aicda*$^{-/-}$ ΔΨVλ B cells complemented with the indicated AID variants-ires-GFP or empty vector (Ctrl). Medians (bars) from 12 cultures/construct from 1 experiment are shown. (**c**) Class switch recombination activity in *Aicda*$^{-/-}$ mouse primary B cells complemented with the indicated AID variants-ires-GFP and stimulated with LPS and IL-4. Mean (bars) proportion of IgG1+ cells in the GFP+ population 72 h after transduction, from 2 independent experiments, with 2 mice per experiment (dots), are shown, normalized to AID. In (**b-d**) WB of cell extracts probed with anti-AID antibody and loading control are shown on the right. For gel source data see supplementary Fig. 7.

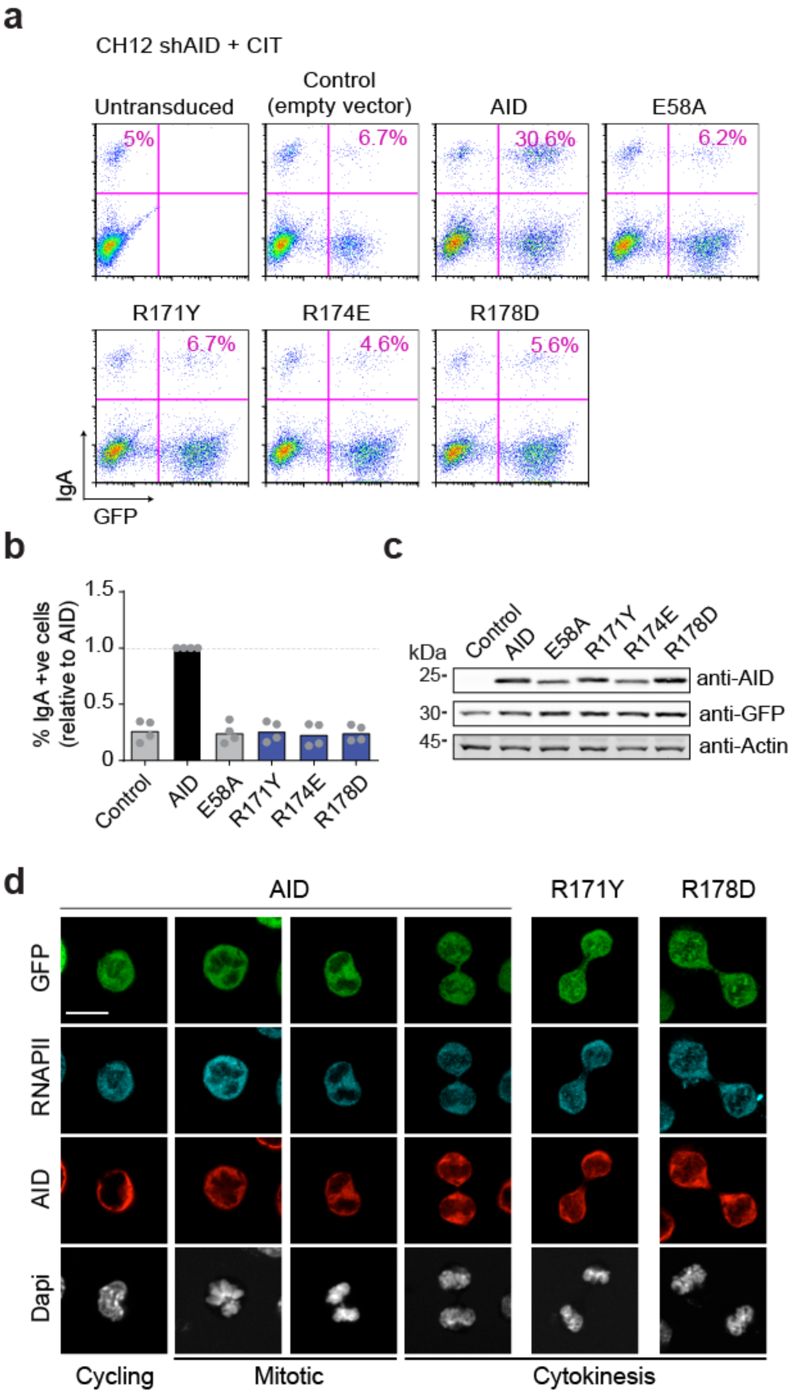

Supplementary Figure 3. Reconstitution of AID-deficient CH12 B cells.
(**a**) CH12 B cells constitutively expressing a shRNA against AID were reconstituted with AID variants by transducing with pMX-AID variant-ires-GFP. Cultures were then stimulated with CIT for 72 h to induce CSR to IgA. Representative flow cytometry plots comparing GFP infection and IgA levels in CIT-stimulated cells. The proportion of GFP+ cells that are IgA+ is indicated. (**b**) Means (bars) of the proportion of GFP+ cells that are IgA+ from 4 independent experiments (dots) are shown, normalized to AID. (**c**) Western blots of extracts from reconstituted AID-deficient CH12 B cells. GFP is used as a control of reconstitution and actin as a loading control. For gel source data see supplementary Fig. 7. (**d**) Confocal microscopy images of CH12 cells analysed by anti-AID and anti-RNAPII IF. Cells were determined to be cycling (G1/S/G2), mitotic or in cytokinesis based on DNA condensation and RNAPII access to the DNA. Images are representative of at least 10 different events per construct from 1 experiment. Magnification 630X. Scale bar, 10 μm.

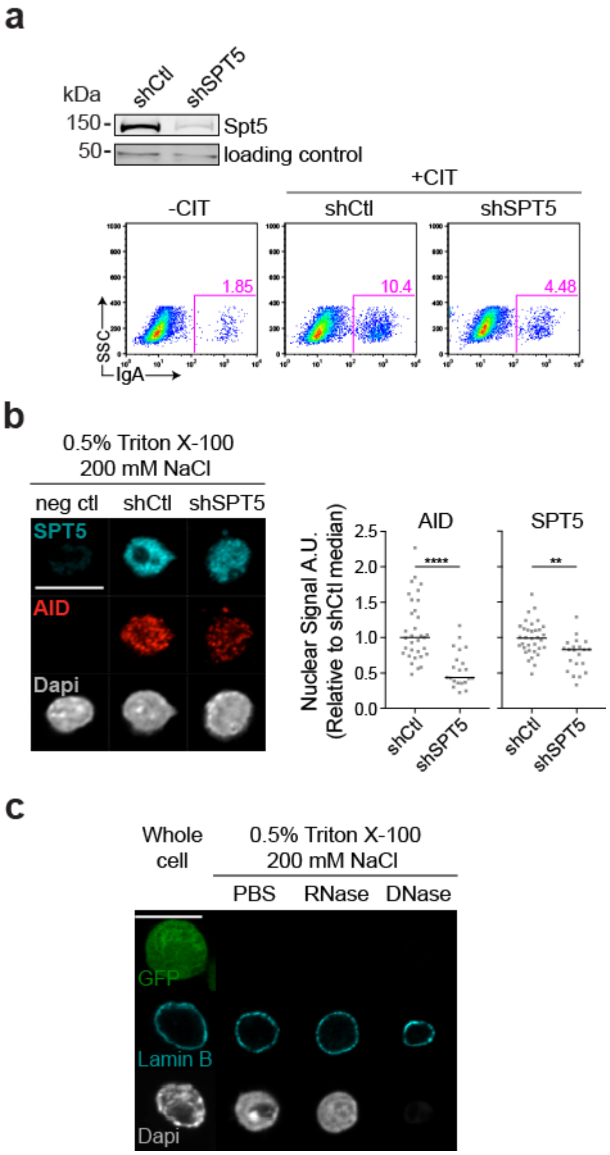

Supplementary Figure 4. AID chromatin association controls

(**a**) (Top) Western blot for Spt5 and loading control (non-specific band) from wt CH12 B cells transduced with shRNA against either luciferase (shCtl) or Spt5. (Bottom) Representative flow cytometry plots showing the proportion of IgA+ cells in unstimulated cells (-CIT) or stimulated cells (+CIT) expressing each shRNA. For gel source data see supplementary Fig. 7. (**b**) CH12 cells expressing each shRNA were stimulated with CIT prior to nuclear wash. (Left) Confocal microscopy images of isolated nuclei analysed by anti-AID and anti-Spt5 (by IF) or DNA (Dapi). (Right) Mean AID or Spt5 signal for each nucleus (dots) and population median (bars). Significant changes in AID or Spt5 signal by unpaired, two tailed t-tests are shown (** <0.01, **** <0.0001). (**c**) Representative confocal microscopy images of GFP, Lamin B (by IF) or DNA (Dapi) on whole cells expressing GFP control or isolated nuclei thereof. During nuclear wash, nuclei were incubated at 37°C with PBS control, RNAse or DNase, as indicated. (**b, c**) Magnification 630X. Scale bar, 10 µm.

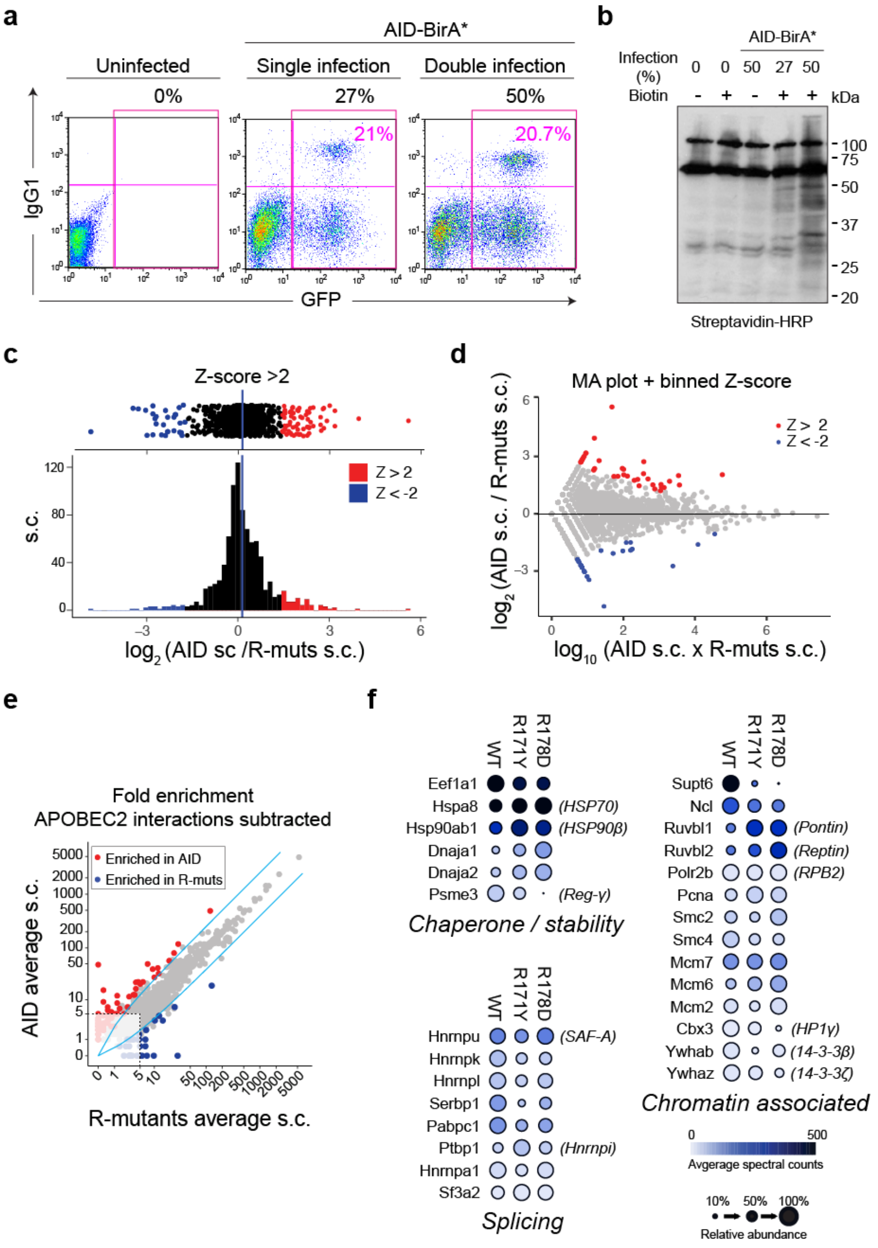

Supplementary Figure 5. BioID controls and alternative statistical methods

(**a**) B cells from *Aicda*$^{-/-}$ mice were transduced once or twice with pMX-AID-BirA*-ires-GFP. Representative flow cytometry plots showing the infection efficiency (above) and the relative proportions of IgG1+ in infected cells. (**b**) Representative western blot probed with streptavidin-HRP to detect biotinylated proteins 24h after adding biotin. Endogenous biotinylation occurs in the cells (lanes 0%), but BirA* dependent signal is only detected when the cells are cultured with biotin (+), and is proportional to the level of infection. For gel source data see supplementary Fig. 7. (**c**) Z-score analysis, with positive Z-score values representing hits enriched for wt AID over R-mutants and negative values representing hits enriched for the R-mutants over wt AID. Positive hits were determined as those ± 2 SD away from the median. (**d**) Hits were distributed based on their overall s.c. for AID and the R-mutants, and then binned in order to run independent Z-score analysis based on overall association. Positive hits were considered as ±2 SDs from the median. (**e**) Comparison of average s.c. for either wt AID or R-mutants interactions after subtracting A2 interactions. Teal lines delimit 2.5 fold changes in enrichment. Dashed box indicates hits with ≤5 s.c., which were excluded from the analysis. Interactions enriched 2.5x in AID over both R-mutants are shown in red, and those 2.5x in R-mutants over AID in blue. (**f**) Dot plot for multiple known AID interactions that were detected by BioID using the method described in (**e**). Proteins were assigned into categories based on their main function. Alternate names for certain proteins are indicated in brackets. Circle size indicates relative abundance normalized to the AID variant with the most s.c., and colour indicates actual s.c. of each factor (scales are included).

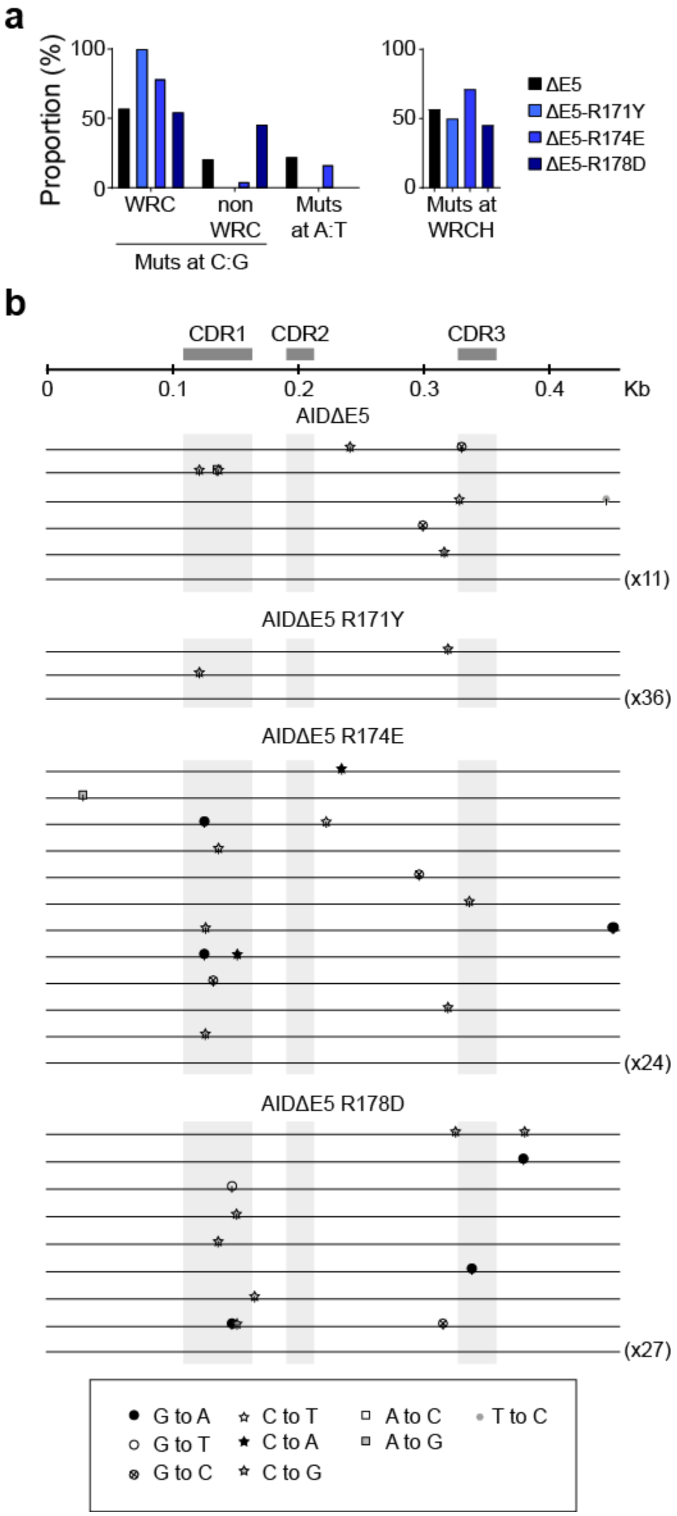

Supplementary Figure 6. SHM at the DT40 IgV by AIDΔE5 R-mutants.

(**a**) Bar plots of proportion of mutations at C:G within WRC (W = A/T, R = A/G) motifs or not, or at A:T pairs (left) or within the AID preferred sequence WRCH (H = A/C/T) (right). (**b**) (Top) Schematic of the IgV region sequenced, with CDR regions highlighted. (Middle) Scheme of individual sequences analyse with mutations indicated (data used for Fig. 8g). CDRs highlighted to indicate the normal distribution of SHM by the R-mutants.

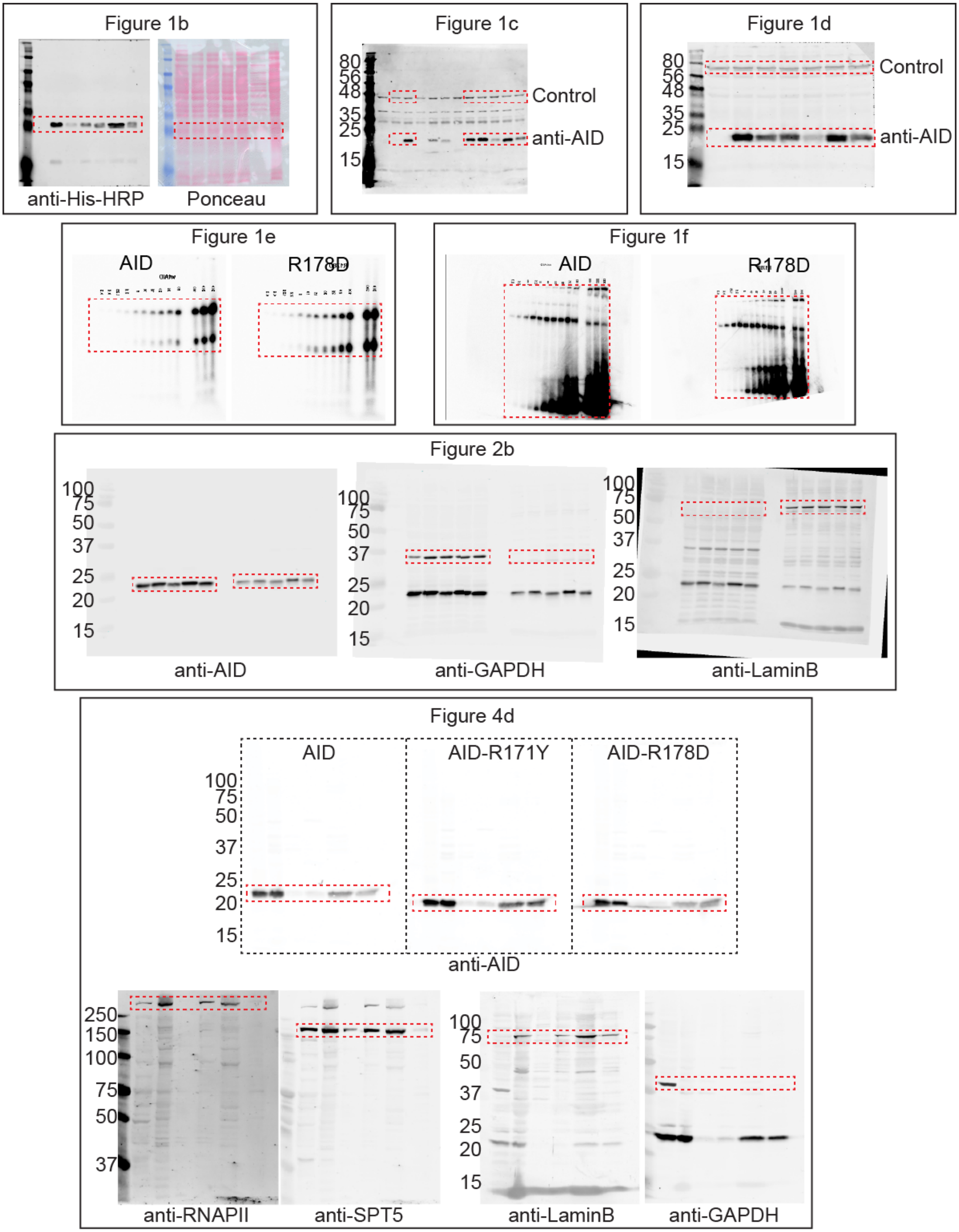

Supplementary Figure S7. Uncropped Western Blots.
Uncropped images of all WBs, with dashed, red boxes indicating cropping used.

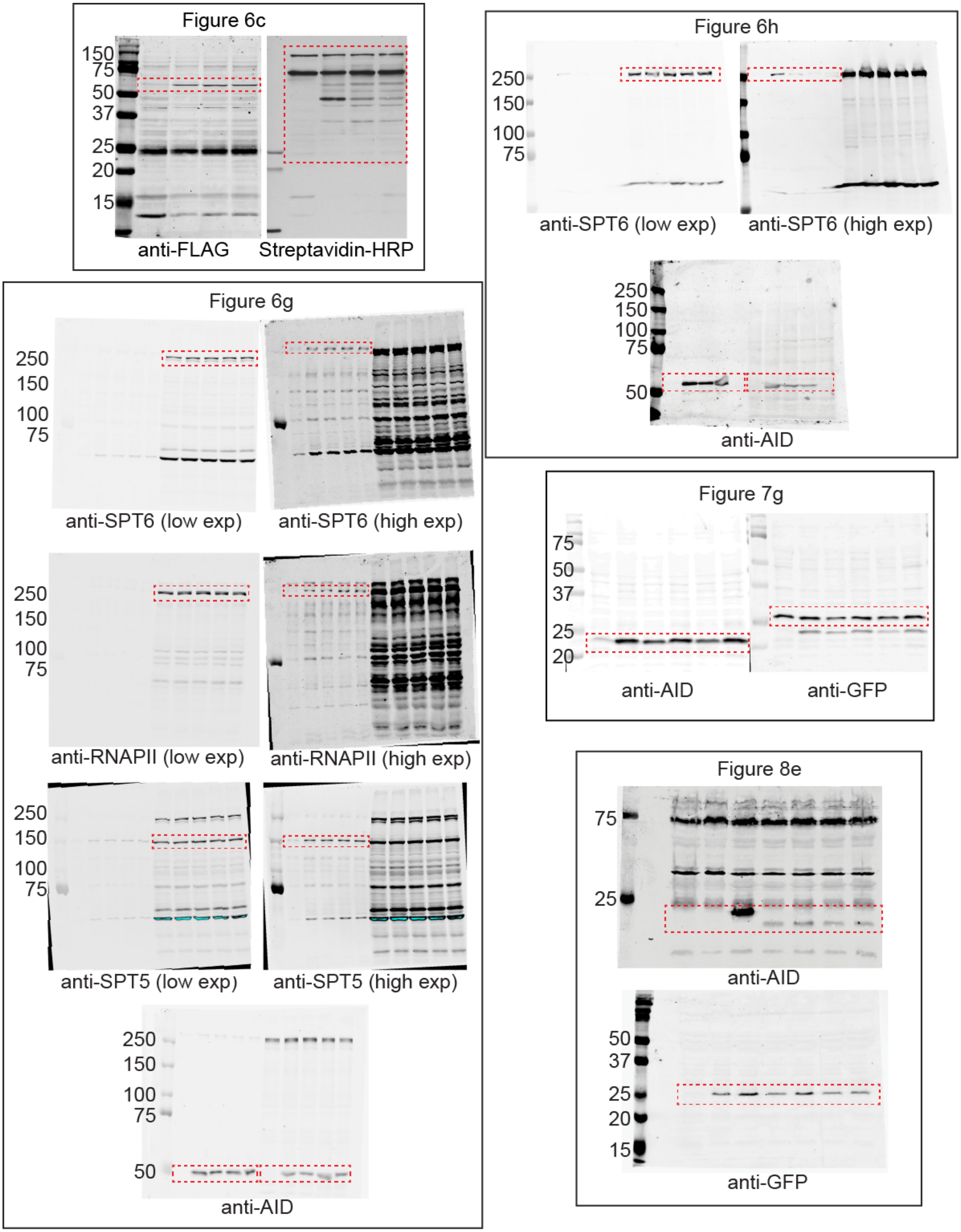

Supplementary Figure S7. Uncropped Western Blots (cont).
Uncropped images of all WBs, with dashed, red boxes indicating cropping used.

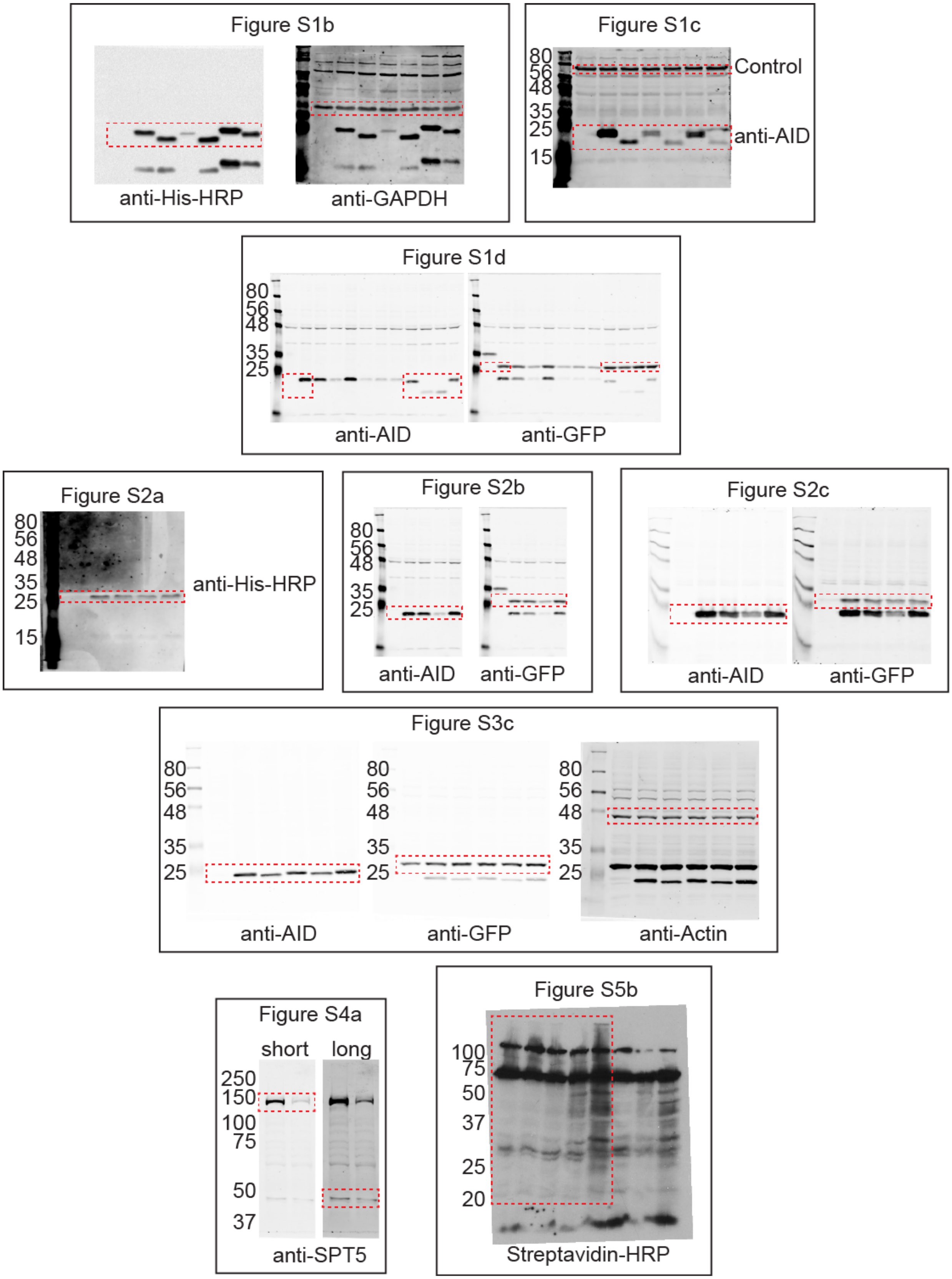

Supplementary Figure S7. Uncropped Western Blots (cont).
Uncropped images of all WBs, with dashed, red boxes indicating cropping used.

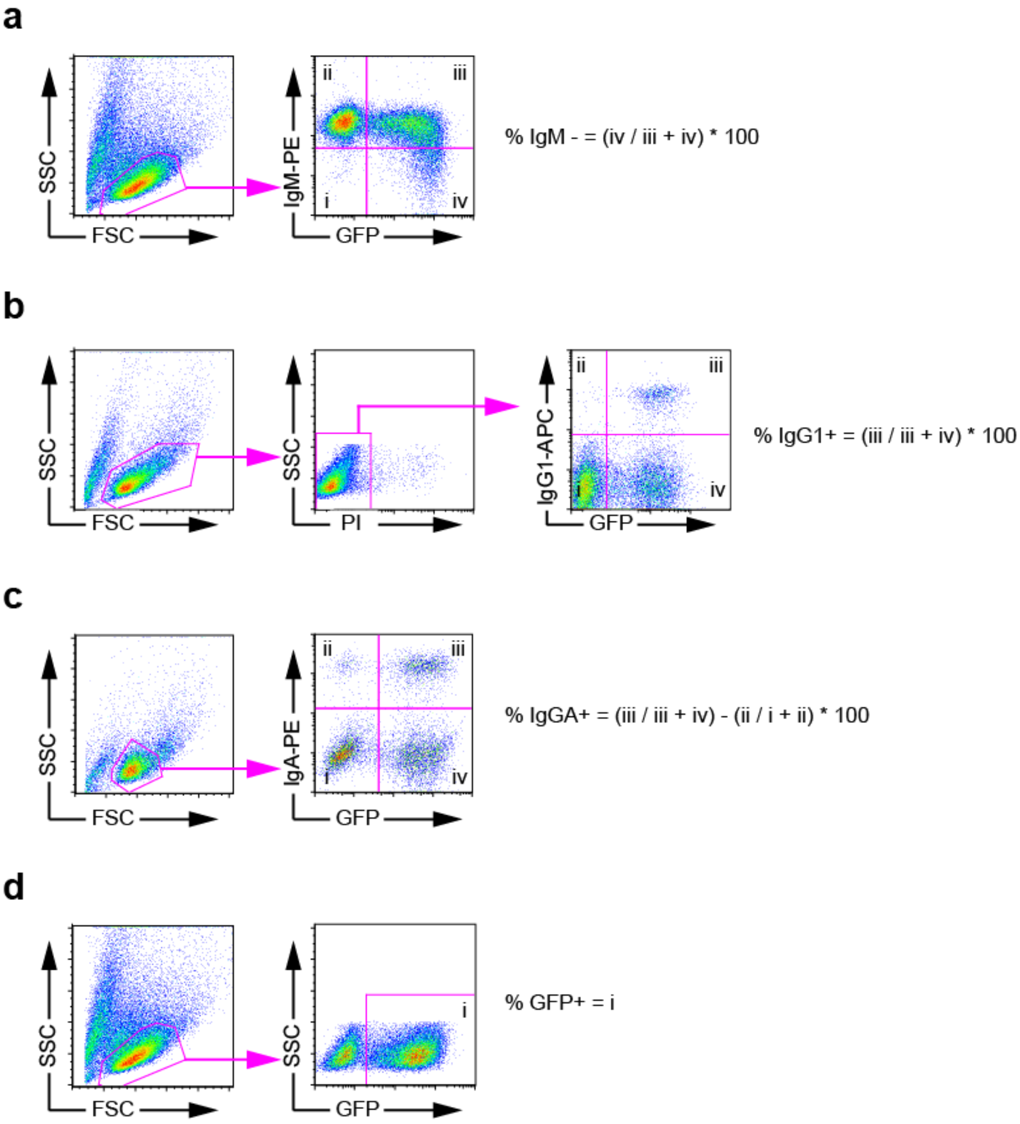

Supplementary Figure S8. Diagram of flow cytometry gating strategies.
Schemes of gating strategies used in various flow cytometry analysis. Magenta arrows demonstrate gates that were further dissected. Roman numerals indicate gates used for analysis, with example calculations indicated on the right. (**a**) Strategy for measuring CSR in primary B cells, used in Fig. 1d, Fig. 6a, b, Supplementary Fig. 1c, Supplementary Fig. 2c. (**b**) Strategy for measuring CSR in CH12 B cells, used in Fig. 3d. (**c**) Strategy for measuring SHM in DT40 B cells, used in Fig. 1c, Fig. 3e, Fig. 8e, Supplementary Fig. 1d and Supplementary Fig. 2b. (**d**) Strategy used for measuring GFP levels in CH12 and DT40 B cells, used in Fig. 3b, f, and Fig. 8c.

Supplementary table 1 - AID BioID interactions reduced in the R mutants according to 4 different methods

	Statistical method*									Spectral counts (s.c.)						s.c. sum		
	1	2	3	4														
Geneid	Fold*	Normz**	Maz***	Deseq	P-value****	Total	genename	Functional category	Function	aid2	aid1	m1712	m1711	m1782	m1781	aid	m171	m178
108723	1	1	1	1	9.59E-02	4	**Card11**	Signaling	BCR signaling	23	18	6	5	7	2	20.5	5.5	4.5
15163	1	1	1	1	3.76E-08	4	**Hcls1**	Signaling	BCR signaling	116	119	27	41	21	29	117.5	34	25
17060	1	1	1	1	1.16E-03	4	**Blnk**	Signaling	BCR signaling	16	15	1	0	0	0	15.5	0.5	0
192119	1	1	1	1	9.63E-02	4	**Dicer1**	RNA processing	Translation silencing	9	9	0	1	0	0	9	0.5	0
20926	1	1	1	1	2.47E-02	4	**Supt6**	Chromatin associated	Histone chaperone / transcription elongation	494	482	142	197	71	64	488	169.5	67.5
223691	1	1	1	1	1.16E-03	4	**Eif3l**	Translation	Translation initiation	37	42	9	9	7	12	39.5	9	9.5
227648	1	1	1	1	2.12E-02	4	**Sec16a**	Protein transport	Protein transport	20	25	3	7	4	4	22.5	5	4
23970	1	1	1	1	6.26E-15	4	**Pacsin2**	Signaling	Signaling	49	47	0	0	0	0	48	0	0
17955	1		1	1	5.17E-02	3	**Nap1l4**	Chromatin associated	Histone chaperone / transcription elongation	24	21	6	6	5	6	22.5	6	5.5
217337	1		1	1	5.06E-03	3	**Srp68**	Protein transport	Protein transport	50	64	25	18	15	21	57	21.5	18
217869	1		1	1	1.69E-04	3	**Eif5**	Translation	Translation initiation / mRNA transport	69	90	18	27	32	21	79.5	22.5	26.5
67154	1		1	1	5.39E-03	3	**Mtdh**	Signaling	Signaling	44	39	11	7	15	14	41.5	9	14.5
13544	1	1	1			3	**Dvl3**	Signaling	Signaling	78	21	0	15	2	12	49.5	7.5	7
17535	1	1	1			3	**Mre11a**	Chromatin associated	DNA damage response	11	13	3	2	0	0	12	2.5	0
19069	1	1	1			3	**Nup88**	Chromatin associated	Nuclear pore / mRNA transport / Transcription silencing	7	10	2	3	0	0	8.5	2.5	0
230721	1	1	1			3	**Pabpc4**	RNA processing	mRNA transport	22	22	10	4	0	2	22	7	1
353258	1	1	1			3	**Ltv1**	Other	Ribosome biogenesis / rRNA processing	9	5	1	1	0	0	7	1	0
110611			1	1	5.11E-02	2	**Hdlbp**	Chromatin associated	RNA binding protein / heterochromatin formation	30	29	12	6	3	13	29.5	9	8
13669			1	1	7.67E-05	2	**Eif3a**	Translation	Translation initiation	113	91	28	31	43	42	102	29.5	42.5
226594			1	1	1.95E-02	2	**Rcsd1**	Other	Actin remodeling	42	45	13	18	17	13	43.5	15.5	15
56347			1	1	2.12E-02	2	**Eif3c**	Translation	Translation initiation / mRNA transport	57	62	29	30	12	21	59.5	29.5	16.5
75705			1	1	3.34E-02	2	**Eif4b**	Translation	Translation initiation / mRNA transport	50	60	12	13	22	37	55	12.5	29.5
110355	1	1				2	**Adrbk1**	Signaling	Signaling	4	6	0	0	0	0	5	0	0
16341	1		1			2	**Eif3e**	Translation	Translation initiation	26	29	6	13	11	10	27.5	9.5	10.5
20383		1	1			2	**Srsf3**	RNA processing	Splicing / mRNA export	8	10	0	1	3	2	9	0.5	2.5
224742	1		1			2	**Abcf1**	Translation	Translation initiation	13	20	3	0	6	7	16.5	1.5	6.5
320528	1	1				2	**Vps13c**	Other	mitochondrial maintenance	9	3	2	0	0	0	6	1	0
58194		1	1			2	**Sh3kbp1**	Signaling	Signaling	6	9	0	0	0	0	7.5	0	0
74112	1	1				2	**Usp16**	Chromatin associated	H2A de-ubiquitinase / transcription elongation	2	9	0	0	2	1	5.5	0	1.5
17886				1	1.16E-03	1	**Myh9**	Other	Cytoskeleton	142	135	66	69	62	78	138.5	67.5	70
208643				1	1.60E-02	1	**Eif4g1**	Translation	Translation initiation / mRNA transport	139	118	50	72	69	84	128.5	61	76.5
226562				1	1.06E-04	1	**Prrc2c**	Other	Unknown	200	169	97	91	69	79	184.5	94	74
27979				1	6.57E-02	1	**Eif3b**	Translation	Translation initiation	52	62	23	22	34	25	57	22.5	29.5
73158				1	1.43E-02	1	**Larp1**	Translation	Translation initiation	107	91	45	50	41	62	99	47.5	51.5
103963	1					1	**Rpn1**	Other	N-oligosaccharyl transferase / Proteasome	14	9	0	3	5	3	11.5	1.5	4
107951	1					1	**Cdk9**	Chromatin associated	Transcription elongation / cyclin dependent kinase	15	10	2	3	2	8	12.5	2.5	5
19192			1			1	**Psme3**	Other	Proteasome	43	43	32	31	3	4	43	31.5	3.5
213988	1					1	**Tnrc6b**	RNA processing	Translation silencing	7	7	3	2	2	3	7	2.5	2.5
218973			1			1	**Wdhd1**	Chromatin associated	Replication initiation	12	17	2	0	0	11	14.5	1	5.5
236732		1				1	**Rbm10**	RNA processing	Splicing	5	5	0	0	0	1	5	0	0.5
27984		1				1	**Efhd2**	Other	Clacium binding	9	3	0	0	0	2	6	0	1
319322		1				1	**Sf3b2**	RNA processing	Splicing	6	4	0	0	0	0	5	0	0
56440			1			1	**Snx1**	Protein transport	Protein transport	20	32	4	0	7	19	26	2	13
59021	1					1	**Rab2a**	Protein transport	Protein transport	7	6	0	2	2	2	6.5	1	2
66085			1			1	**Eif3f**	Translation	Translation initiation	45	55	26	11	30	20	50	18.5	25
66448	1					1	**Mrpl20**	Other	Mitochondrial ribosome	6	4	4	0	0	0	5	2	0
67062	1					1	**Slc25a53**	Protein transport	Mitochondrial transport	5	5	0	1	4	0	5	0.5	2
67166	1					1	**Arl8b**	Signaling	GTPase	6	4	3	1	2	2	5	2	2
67543	1					1	**Pabpc6**	RNA processing	mRNA transport	30	35	0	26	23	0	32.5	13	11.5
68926	1					1	**Ubap2**	Other	Protein ubiquitination	14	11	1	9	4	0	12.5	5	2
69710		1				1	**Arap1**	Protein transport	Protein transport	4	7	0	0	0	0	5.5	0	0
72567		1				1	**Bclaf1**	Chromatin associated	Transcriptional repressor	5	6	0	0	0	0	5.5	0	0
75786		1				1	**Ckap5**	Other	Cytoskeleton	6	8	0	0	0	3	7	0	1.5
76302		1				1	**Pcnp**	Other	Cell cycle	6	6	2	2	0	0	6	2	0

* Preys enriched by >2.5 fold s.c. in AID over R-muts average AND 5-fold over APOBEC2 s.c. for the same prey

** Preys with Z-score >2 were considered differential interactions.

*** Preys with local Z-score >2 over a sliding window including 10% of the points around the candidate were considered differential interactions.

**** P-values calculated from negative binomial distributions of the data.

Supplementary table 2 - Oligonucleotides

ChIPs in mouse splenic B cells

Amplicon name	Target site		Sequence	Reference
P-a	Sμ TSS a	fwd	CCACCTGGGTAATTTGCATTTC	This study
		rev	GGGAAACTAGAACTACTCAAGCTAA	
P-b	Sμ TSS b	fwd	AGCTTGAGTAGTTCTAGTTTCCC	This study
		rev	GAGACCAATAATCAGAGGGAAGAA	
Sμ-a	Sμ upstream from repeats	fwd	TAGTAAGCGAGGCTCTAAAAAGCAC	Cortizas et al. 2013
		rev	ACTCAGAGAAGCCCACCCAT	
Sμ-b	Sμ downstream from repeats	fwd	GGTTGGGAGACCATGAATTG	Cortizas et al. 2013
		rev	TTCTTAGCTCAACCCAGTTTATCC	
Cμ	Cμ	fwd	CTGAACCTGAGGGAGTCAGC	Cortizas et al. 2013
		rev	GCCACTGCACACTGATGTCT	
P	Sγ1 TSS	fwd	GCTGCAAGAAGAGGCCATAC	This study
		rev	CTCCTTCCCAATCTCCCGTG	
Sγ1-a	Sγ1 upstream from repeats	fwd	GAGGAGTGCAGGAAGTCTGG	This study
		rev	CCTTGATGCCCTCCCTTT	
Sγ1-b	Sγ1 downstream from repeats	fwd	GGATGTCTAGGCTGGAGCTG	This study
		rev	GAAGCTCAGGCCTGTTGCTG	
Sα1-a	Sα upstream from repeats	fwd	AAGCAGGCCTGGGGTGGACA	This study
		rev	AGCAAGCTCAGCCCAGCCTAA	
Sα1-b	Sα downstream from repeats	fwd	CTTGGCTAGGCTACAATGGATTGAGC	Cortizas et al. 2013
		rev	GTGCAACTCTATCTAGGTCTGCCCGGT	
Gapdh	*Gapdh ~2.5 Kb downstream from TSS*	fwd	CACCTTCAGCTTTCCGGCCACTTAC	Cortizas et al. 2013
		rev	GGAAGCCCATCACCATCTTCCAGGA	
P	*Gapdh* TSS	fwd	GAGCTACGTGCACCCGTAAA	This study
		rev	AATGAGGCGGGTCCAAAGAG	
P	*Il4ra* TSS	fwd	CCGCAGTCGTGGGACTTAAA	This study
		rev	CAGCTCATTTTCACCCGCAG	
Il4ra 500bp	*Il4ra* 500bp downstream from TSS	fwd	AGTCTGTGATTCCGTCTGCC	This study
		rev	CCACATTGAGCCTCACACCT	
Il4ra 800bp	*Il4ra* 800bp downstream from TSS	fwd	GACAGGGAGTGTATTGGGCA	This study
		rev	GTTCTCCCACACCCCAAACT	

ChIPs in DT40 cells

Amplicon name	Target site		Sequence	Reference
P	IgV TSS	fwd	CGGAAGGACGCGGGTATAAA	Romanello et al. 2016
		rev	ACCAGGCGCAACGAGTAC	
IgVλ	IgV 400bp downstream from TSS	fwd	CCCTTCACGATTCTCCGGTT	Romanello et al. 2016
		rev	GTCAGCGACTCACCTAGGAC	
intron	IgV intron	fwd	TGGTCTCTCACTGGGGACTC	Romanello et al. 2016
		rev	GCACTTACCTGGACAGCTGA	
GAPDH	DT40 *GAPDH*	fwd	TGTTTGTGATGGGTGTCAAC	Romanello et al. 2016
		rev	GCATTGCTGGGAAAGAAAGAAG	

Primers used for sequencing mutations

Figure	Target site		Sequence	Reference
Fig. 3a	Sμ TSS to S region (mouse)	fwd	AGCTTGAGTAGTTCTAGTTTCCC	This study
		rev	CAGTCCAGTGTAGGCAGTAGA	Zahn et al. 2014
Fig. 8f	IgVλ (DT40)	fwd	CAGGAGCTCGCGGGGCCGTCACTGATTGCCG	Sale et al. 2001
		rev	GCGCAAGCTTCCCCAGCCTGCCGCCAAGTCCAAG	
Fig. 8g	Sμ S region (mouse)	fwd	GTAAGGAGGGACCCAGGCTAAG	Zahn et al. 2014
		rev	CAGTCCAGTGTAGGCAGTAGA	

Quickchange primers

Mutation	Protein		Sequence	
R171Y	human AID	fwd	GAAGGGCTGCATGAAAATTCAGTTTATCTCTCCAGACAGCTT	This study
		rev	AAGCTGTCTGGAGAGATAAACTGAATTTTCATGCAGCCCTTC	
S173E	human AID	fwd	CATGAAAATTCAGTTCGTCTCGAGAGACAGCTTCGGCGCATCCTT	This study
		rev	AAGGATGCGCCGAAGCTGTCTCTCGAGACGAACTGAATTTTCATG	
R174E	human AID	fwd	GAAAATTCAGTTCGTCTCTCCGAGCAGCTTCGGCGCATCCTTTTG	This study
		rev	CAAAAGGATGCGCCGAAGCTGCTCGGAGAGACGAACTGAATTTTC	
R177A	human AID	fwd	GTTCGTCTCTCCAGACAGCTTGCGCGCATCCTTT	This study
		rev	AAAGGATGCGCGCAAGCTGTCTGGAGAGACGAAC	
R178D	human AID	fwd	CTCCAGACAGCTTCGGGACATCCTTTTGCCCCTG	This study
		rev	CAGGGGCAAAAGGATGTCCCGAAGCTGTCTGGAG	
R171K	human AID	fwd	CTGGGAAGGGCTGCATGAAAATTCAGTTAAGCTCTCCAGACAGCTT	This study
		rev	AAGCTGTCTGGAGAGCTTAACTGAATTTTCATGCAGCCCTTCCCAG	
R174K	human AID	fwd	CAGTTCGTCTCTCCAAACAGCTTCGGCGC	This study
		rev	GCGCCGAAGCTGTTTGGAGAGACGAACTG	
R178K	human AID	fwd	CATACAGGGGCAAAAGGATCTTCCGAAGCTGTCTGGAGAGA	This study
		rev	TCTCTCCAGACAGCTTCGGAAGATCCTTTTGCCCCTGTATG	
E58A	human AID	fwd	CGGCTGCCACGTGGCATTGCTCTTCCTCCG	Zahn et al. 2014
		rev	CGGAGGAAGAGCAATGCCACGTGGCAGCCG	
R171Y	mouse AID	fwd	GCGCCGAAGTTGTCTGGTTAGATAGACAGAATTTTCATGTAGCCC	This study
		rev	GGGCTACATGAAAATTCTGTCTATCTAACCAGACAACTTCGGCGC	
R174E	mouse AID	fwd	CAAAAGGATGCGCCGAAGTTGCTCGGTTAGCCGGACAGAATTTTC	This study
		rev	GAAAATTCTGTCCGGCTAACCGAGCAACTTCGGCGCATCCTTTTG	
R178D	mouse AID	fwd	GTACAAGGGCAAAAGGATGTCCCGAAGTTGTCTGGTTAGC	This study
		rev	GCTAACCAGACAACTTCGGGACATCCTTTTGCCCTTGTAC	

Supplementary table 2 - Oligonucleotides (Cont.)

Cloning primers (restriction sites removed)

Construct	Protein		Sequence	
AID	human AID	fwd	ATGGACAGCCTCTTGATGAA	This study
		rev	TCAAAGTCCCAAAGTACGAAA	
AIDΔE5	human AIDΔE5	fwd	ATGGACAGCCTCTTGATGAA	This study
		rev	TCACAAAAGGATGCGCCGAAG	
AIDΔE5-R178D	human AIDΔE5	fwd	ATGGACAGCCTCTTGATGAA	This study
		rev	CTACAAAAGGATGTCCCGAAGCTGTCTGGAGAGACG	
AID	mouse AID	fwd	ATGGACAGCCTTCTGATGAA	This study
		rev	TCAAAATCCCAACATACGAAATGCA	

Gateway cloning primers (Recombination sequences underlined)

Construct	Protein		Sequence	
acR-mCherry-NLS-SPT	human SPT5	fwd	GGGGACAAGTTTGTACAAAAAAGCAGGCTTCATGTCGGACAGCGAGGACAG	This study
		rev	GGGGACCACTTTGTACAAGAAAGCTGGGTTCTAGGCTTCCAGGAGCTTCCCCA	
AID-Linker-BirA*-flag	mouse AID	fwd	GGGGACAAGTTTGTACAAAAAAGCAGGCTTCACCATGGACAGCCTTCTGATGAAGCA	This study
		rev	GGGGACAACTTTTGTATACAAAGTTGTAAATCCCAACATACGAAATGCATCT	
AID-Linker-BirA*-flag	Linker	fwd	GGGGACAACTTTGTATACAAAAGTTGTAGGTGGCGGAGGGAGTGGAGGCGGTGGCAGC	This study
AID-Linker-BirA*-flag	Linker-BirA*-flag	fwd	TGGAGGCGGTGGCAGCGGTGGCGGAGGGAGTATGAAGGACAACACCGTGCC	This study
		rev	GGGGACCACTTTGTACAAGAAAGCTGGGTTCTATTTATCGTCATCGTCTTTGTAGTCT	
APOBEC2-BirA*-flag	human APOBEC2	fwd	GGGGACAAGTTTGTACAAAAAAGCAGGCTTCATGGCCCAGAAGGAAGAGGCT	This study
		rev	GGGGACAACTTTTGTATACAAAGTTGTCTTCAGGATGTCTGCCAACTTCT	
APOBEC2-BirA*-flag	BirA*-flag	fwd	GGGGACAACTTTGTATACAAAAGTTGTAATGAAGGACAACACCGTGCC	This study
		rev	GGGGACCACTTTGTACAAGAAAGCTGGGTTCTATTTATCGTCATCGTCTTTGTAGTCT	

Modular AID fusion constructs (restriction sites underlined)

Construct	Protein		Sequence	
AID-linker-AID(E58A)	N-term human AID	fwd	nnGGATCCaccATGGACAGCCTCTTGATGAA	This study
		rev	cGAATTCccAAGTCCCAAAGTACGAAATGC	
AID-linker-AID(E58A)	Linker	sense	AATTCGGTGGCGGAGGGAGTGGAGGCGGTGGCAGCGGTGGCGGAGGGAGTA	This study
		antisens	AGCTTACTCCCTCCGCCACCGCTGCCACCGCCTCCACTCCCTCCGCCACCG	
AID-linker-AID(E58A)	C-term human AID	fwd	nnnAAGCTTATGGACAGCCTCTTGATGAA	This study
		rev	nnnnCTCGAGTCAAAGTCCCAAAGTACGAAA	

Supplementary references

1. Cortizas, E.M. et al. Alternative End-Joining and Classical Nonhomologous End-Joining Pathways Repair Different Types of Double-Strand Breaks during Class-Switch Recombination. *J Immunol* **191**, 5751-63 (2013).
2. Romanello, M., Schiavone, D., Frey, A. & Sale, J.E. Histone H3.3 promotes IgV gene diversification by enhancing formation of AID-accessible single-stranded DNA. *The EMBO journal* **35**, 1452-1464 (2016).
3. Zahn, A. et al. Activation induced deaminase C-terminal domain links DNA breaks to end protection and repair during class switch recombination. *Proceedings of the National Academy of Sciences of the United States of America* **111**, E988-97 (2014).
4. Sale, J.E., Calandrini, D.M., Takata, M., Takeda, S. & Neuberger, M.S. Ablation of XRCC2/3 transforms immunoglobulin V gene conversion into somatic hypermutation. *Nature* **412**, 921-6 (2001).
